# A potential role for AHR in SARS-CoV-2 pathology

**DOI:** 10.21203/rs.3.rs-25639/v1

**Published:** 2020-04-27

**Authors:** Federico Giovannoni, Zhaorong Li, Cybele C. Garcia, Francisco J. Quintana

**Affiliations:** Brigham and Women’s Hospital, Harvard Medical School; Universidad de Buenos Aires; Broad Institute of MIT and Harvard

**Keywords:** coronavirus, aryl hydrocarbon receptor activation, lung basal cells

## Abstract

Coronavirus infection is associated to life-threatening respiratory failure. The aryl hydrocarbon receptor (AHR) was recently identified as a host factor for Zika and dengue viruses; AHR antagonists decrease viral titers and ameliorate ZIKV-induced pathology *in vivo*. Here we report that AHR is activated during coronavirus infection, impacting anti-viral immunity and lung basal cells associated to tissue repair. Hence, AHR antagonists are candidate therapeutics for the management of coronavirus-infected patients.

## Introduction, Results And Discussion

Coronaviruses (CoVs) are positive sense single-stranded RNA viruses of major agricultural and public health importance^1^. Coronaviruses were considered of low risk to humans until 2002, when a severe acute respiratory syndrome (SARS) outbreak occurred in Guangdong, China^2–5^. Ten years later, the highly pathogenic Middle East respiratory syndrome coronavirus (MERS-CoV) emerged in the Middle East^6^. In December, 2019, an epidemic of coronavirus disease 2019 (COVID-19) caused by a severe acute respiratory syndrome coronavirus 2 (SARS-CoV-2) originated in Wuhan, China^7,8^. The common symptoms of SARS- CoV-2 infection at onset are fever, fatigue, dry cough, myalgia, and dyspnea^9^. In^5–15^ % of the infected patients, the acute form of the disease causes life-threatening progressive respiratory failure^8,10,11^. The high rate of transmission of SARS-CoV-2 translates into an overwhelming number of patients needing intensive care support, putting an enormous stress on national health systems around the globe. However, no specific therapeutic agents or vaccines are available for COVID-19. Thus, there is an urgent unmet clinical need for candidate targets to treat and prevent SARS-CoV-2 infection.

The ligand-activated transcription factor aryl hydrocarbon receptor (AHR) controls multiple aspects of the immune response^12,13^. AHR activation by metabolites produced by tumors^14,15^ or in the context of viral infection^16^ interferes with the generation of protective immunity. Indeed, AHR suppresses the production of type I interferons (IFN-I)^17,18^, probably as part of a feedback negative mechanism because IFN-I induce AHR expression^19^. We recently showed that AHR activation during the infection with Zika or dengue virus suppresses IFN-I- dependent and IFN-I-independent anti-viral innate and intrinsic immunity^17^. Most importantly, an AHR antagonist optimized for human use boosted anti-viral immunity, interfered with viral replication and ameliorated multiple aspects of Zika congenital syndrome including microcephaly in animal models^17^, identifying AHR as a candidate target for therapeutic intervention. Based on these findings and the urgent need for therapies for SARS-CoV-2, we investigated the potential role of AHR in coronavirus infection.

**Figure 1:**
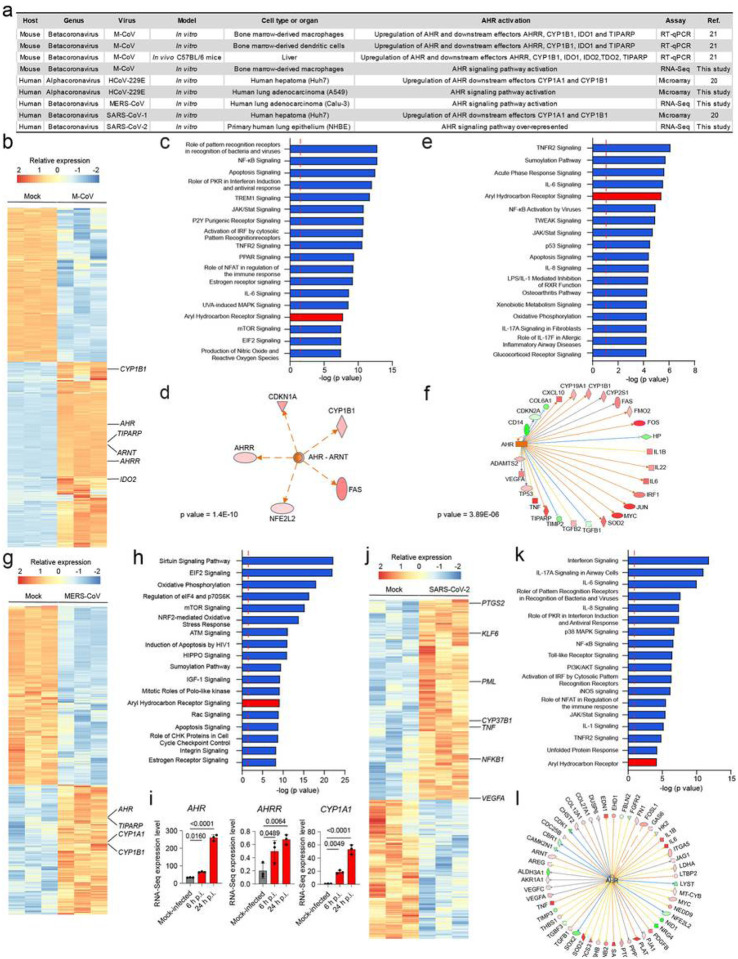
AHR signaling is associated to infection with multiple coronaviruses. (a) Activation of AHR signaling upon infection with different members of the Alphacoronavirus and Betacoronavirus genus of the Coronaviridae family (b) Heatmap showing gene expression detected by RNA-seq analysis of mock-infected and M-CoV-infected bone marrow-derived macrophages (n=3 independent experiments per condition) (c) Ingenuity pathway analysis (IPA) of pathways enriched in M-CoV-infected cells compared to mock-infected cells (n=3 independent experiments per condition). Dashed red line indicates p=0.05. p values were determined using a Fisher’s exact test. (d) IPA identifies AHR as an upstream regulator. p value was determined using a Fisher’s exact test. (e) IPA of pathways enriched in HCoV-229E- infected cells compared to mock-infected human lung adenocarcionma (A549) cells (n=3 independent experiments per condition). Dashed red line indicates p=0.05 (f) IPA identifies AHR as an upstream regulator in the infected samples. p value was determined using a Fisher’s exact test. (g) Heatmap showing gene expression detected by RNA-seq analysis of mock-infected and MERS-CoV-infected human lung adenocarcinoma (Calu-3) cells (n=3 independent experiments per condition) (h) IPA of pathways enriched in MERS-CoV-infected cells compared to mock- infected cells (n=3 independent experiments per condition). Dashed red line indicates p=0.05. p values were determined using a Fisher’s exact test. (i) Expression levels of AHR, AHRR and CYP1A1 determined at different times post infection by RNA-Seq (n=3 independent experiments per condition) (j) Heatmap showing gene expression detected by RNA-seq analysis of mock-infected and SARS-CoV-2-infected human primary lung epithelium cells (n=3 independent experiments per condition) (k) IPA of pathways enriched in SARS-CoV-2-infected cells compared to mock-infected cells (n=3 independent experiments per condition). Dashed red line indicates p=0.05. p value was determined using a Fisher’s exact test. (l) IPA identifies AHR as an upstream regulator in the infected samples. p value determined using a Fisher’s exact test.

Early studies using gene expression microarrays analyzed the transcriptional response to infection by multiple coronaviruses, including the murine coronavirus (M-CoV) of the betacoronavirus genus, and the human coronavirus 229E (HCoV-229E) usually associated to common cold. We detected increased expression of the AHR transcriptional targets *CYP1A1* and *CYP1B1* in response to M-CoV and HCoV-229E infection (Fig. 1a). These findings were confirmed by a recent study which analyzed the transcriptional response to M-CoV infection *in vitro* and *in vivo*^20^. We also detected the activation of AHR signaling in M-CoV^21^, HCoV-229E^22^, MERS-CoV^23^, SARS-CoV-1^20^ and SARS-CoV-2^24^ gene expression data available in the Gene Expression Omnibus (GEO) public database (Fig. 1a).

In depth analyses of RNA-Seq data from M-CoV infected bone marrow-derived macrophages detected the up-regulation of AHR and related genes such as *IDO2*, *CYP1B1*, *AHRR* and *TIPARP* (Fig 1b). IDO2 catalyzes the production of AHR agonists in the context of tumors^25^ and viral infections^17,26^, and TIPARP contributes to the suppression of IFN-I expression^18^. Ingenuity pathway analysis (IPA) detected the enrichment of pattern recognition receptors and immune cell signaling molecules involved in antiviral IFN-related mechanisms, including NF-κB, JAK/Stat, PKR, IRF and IL-6, as well as a significant enrichment in AHR signaling (Fig. 1c). Moreover, upstream analysis identified AHR-ARNT as candidate regulators of the transcriptional response to M-CoV infection. These findings suggest that AHR participates in the transcriptional response of M-CoV infected cells (Fig. 1d).

Next, we analyzed a dataset of HCoV-229E infected A549 cells; IPA analysis detected AHR among the most highly enriched pathways in infected cells. Moreover, we identified AHR as a regulator of the transcriptional response of infected samples (Figs. 1e,f). The analysis of RNA-seq data from MERS-CoV infected human lung adenocarcinoma (Calu-3) cells detected the up-regulation of *AHR* and related genes (*CYPA1*, *CYP1B*1 and *TIPARP*) (Fig. 1g). Accordingly, IPA detected the activation of a broad range of cellular processes, including AHR signaling (Fig. 1h). Of note, *AHR*, *AHRR* and *CYP1A1* expression determined by RNA-seq was found to be gradually up-regulated at different times post infection (Fig. 1i). Finally, we analyzed RNA-seq data of mock-infected and SARS-CoV-2 infected human primary lung epithelium cells. IPA of differentially expressed genes in SARS-CoV-2 infected cells compared to mock-infected cells detected the activation of the AHR pathway, together with IFN signaling, NF-KB, JAK/Stat and others (Figs. 1j,k). In addition, AHR was also identified as an upstream regulator of relevant cytokines and chemokines involved in the response to viral infection and in ammation-related cellular processes (Fig. 1l).

**Figure 2:**
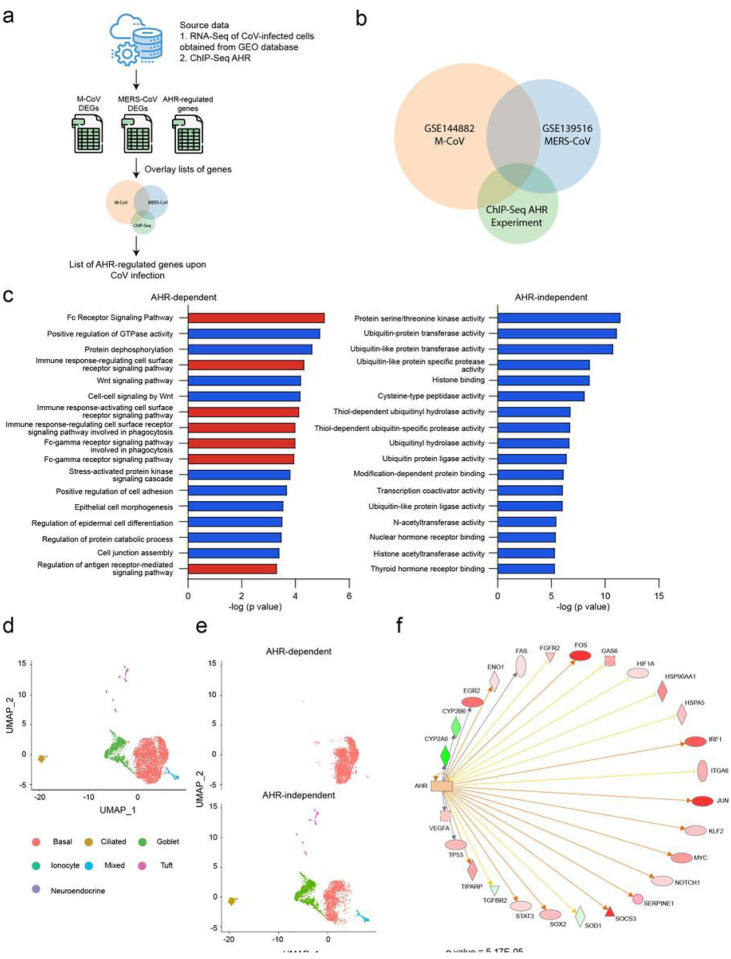
Identification of an AHR-dependent module in the transcriptional response to coronavirus infection. (a) Strategy used to identify AHR-dependent modules in the transcriptional response to coronavirus infection. Significantly up-regulated genes in each RNA- seq dataset were overlapped with genes associated to peaks identified by AHR ChIP-seq within 1 kilobase distance to the transcription start region. (b) Venn diagram representing the overlap in significantly up-regulated genes in M-CoV-infected cells, MERS-CoV-infected cells and the ones identified by an AHR ChIP-Seq experiment. (c) Pathway enrichment analysis was performed on two gene sets: the one resulting from the overlap of the three datasets in (b) (“AHR”) and the one resulting from the overlap between the M-CoV and MERS-CoV datasets in (b) (“non-AHR”). p value was determined using a Fisher’s exact test. Pathways colored in red are related to immunity (d) Single cell RNA-sequencing data of mouse trachea epithelia cells. Each cell is labelled by the cell type. (e) “AHR-dependent” and a “AHR-independent” cell populations identified by gene set enrichment analysis using the previously generated gene set (f) Upstream regulator analysis on the AHR activated cell population compared to the non-AHR activated cell population identified AHR as an upstream regulator.

We used a dataset of AHR targets identified in genome-wide ChIP-seq studies to define the AHR-dependent module in the transcriptional response to coronavirus, focusing on M-CoV and MERS-CoV for which the available datasets were most complete (Figs. 2a,b). The pathway enrichment analysis of the AHR-dependent and the AHR-independent components of the transcriptional response to coronavirus infection detected an enrichment in biological pathways related to the immune response and Fc signaling in the AHR-dependent transcriptional module (Fig. 2c).

In about 5–15% of infected patients, SARS-CoV-2 infection causes life-threatening respiratory complications^10,11^. To investigate potential AHR-dependent mechanisms that may contribute to the pathogenesis of COVID-19, we analyzed a single-cell RNA-Seq (scRNA-seq) dataset of lung epithelial cells, identifying six cell populations corresponding to basal cells, goblet cells, ciliated cells, Tuft cells, neuroendocrine cells and pulmonary ionocytes (Fig. 2d). Strikingly, the AHR-dependent transcriptional module induced by coronavirus infection was mostly associated to basal cells (Fig. 2e), which contribute to lung regeneration after multiple types of injuries including influenza infection^27^. Interestingly, IPA analysis of the scRNA-seq dataset of lung epithelial cells identified AHR as a transcriptional regulator of the basal cell cluster (Fig. 2f). These findings suggest that AHR signaling triggered by coronavirus infection interferes with the regenerative activity of lung epithelial basal cells.

In summary, we identified AHR signaling as a common host response to infection by multiple coronaviruses. It has been reported that although some NF-κB signaling is needed for coronavirus replication, excessive activation of this pathway may be deleterious for the virus^22^. AHR limits NF- B activation, and interferes with multiple anti-viral immune mechanisms including IFN-I production and intrinsic immunity^17,18^. Thus, our findings suggest that the modulation of NF-kB signaling via AHR may dampen the immune response against coronavirus. We also detected a potential role of AHR in the control Fc receptor expression and signaling. Based on recent reports on the association of high antibody titers against SARS-CoV-2 with worst clinical outcomes^28^, these findings suggest a role for antibody enhancement in COVID-19 pathogenesis.

Our studies also suggest that AHR signaling associated to coronavirus infection affects lung basal cells, which give rise to stem cells involved in lung repair in multiple contexts including influenza virus infection^29–31^. Of note, AHR-deficient mice show enhanced repair of the lung bronchiolar epithelium following naphthalene injury^32^, concomitant with the increase proliferation and the earlier activation of basal cells. Taken together, these findings suggest that AHR signaling associated to coronavirus infection may interfere with the activity of basal cells, contributing to the lung pathogenesis associated to SARS-CoV-1, MERS-CoV and SARS-CoV-2 infection. Of note, although lung basal cells do not constitutively express ACE2, the cellular entry receptor for SARS-CoV-1^33^ and SARS-CoV-2^8,27^, IFN-I drive ACE2 expression on primary human upper airway basal cells^34^. Thus, AHR signaling may be induced in basal cells following their infection, or indirectly via the up-regulation of enzymes involved in the production of AHR agonists in other cells. Indeed, TDO and IDO2 expression is up-regulated in response to viral infection^17^, probably as part of a mechanism that limits immunopathology^35^ but is exploited by pathogens to evade the immune-response. Most importantly, AHR antagonists activate anti-viral immunity, decrease viral titers and virus-induced pathology in the context of Zika and dengue virus infection^17^. Thus, AHR antagonists developed for clinical use may provide novel approaches for the treatment of COVID-19 patients.

## Methods

### RNA-sequencing alignment and quantification.

The raw fastq les for all samples were downloaded and aligned to Human (GRCh38) and Mouse (GRCm38) reference genome using STAR v2.7.3a^36^. Then the aligned reads were quantified using Rsem v1.3.1^37^.

### Differential expression analysis.

The count matrix was built using the Rsem output for each sample, and then DESeq2^38^ was used to conduct differential expression analysis. The log2 fold change in the results was shrunk using Apeglm^39^.’

### Downstream analysis.

Differentially expressed genes were further analyzed using GSEA^40^ and IPA in order to find enriched pathways and upstream regulators.

### Overlap between the ChIP-seq and bulk RNA-sequencing results.

The list of AHR target genes, generated from a ChIP-seq dataset^41^, was overlapped with the lists of differentially expressed genes from M-CoV and MERS-CoV-infected samples using the threshold of log2 fold change larger than 0 and adjusted p value smaller than 0.05. Then the results were further overlapped to generate a list of up-regulated genes common to all virus-infected samples and ChIP-seq identified AHR targets.

### Download and processing of data.

The single cell dataset was downloaded from the GEO repository (GSE103354). The 10X format count matrices were downloaded and processed using Seurat^42^ including normalization, dimension reduction and clustering. Then for each cluster the GSEA was used to analyze the differential expression analysis results of each cluster with the AHR activation signature generated in the previously. The clusters that had significant up- regulation in AHR activation signature (FDR < 0.25) are marked as “AHR-dependent” and other clusters are marked as “AHR-independent”. Then MAST^43^ was used to conduct the differential expression analysis between the AHR activated population and non AHR activated population.

### Downstream analysis.

Differentially expressed genes were further analyzed using GSEA and IPA in order to find enriched pathways and upstream regulators.

### Cell-trajectory and pseudo-time analysis.

The single cell dataset was further analyzed using monocle^44^. The pseudo-time order of the cells were constructed using the genes that were differentially expressed (p adjusted value < 0.01) between the AHR and the non-AHR activated population.

### Data availability.

The authors declare that the raw data supporting the findings of this study are publicly available. Transcriptomics data (RNA-Seq or microarray) from virus-infected samples, including M-CoV, HCoV-229E, MERS-CoV and SARS-CoV-2 were accessed at GSE144882, GSE89167, GSE139516, and GSE147507 respectively. Trachea epithelial single cell data was accessed at GSE103354.
